# β-Carotene Status Is Associated with Inflammation and Two Components of Metabolic Syndrome in Patients with and without Osteoarthritis

**DOI:** 10.3390/nu13072280

**Published:** 2021-06-30

**Authors:** Chi-Hua Yen, Po-Sheng Chang, Ching-Ju Chiu, Yu-Yun Huang, Ping-Ting Lin

**Affiliations:** 1 School of Medicine, Chung Shan Medical University, Taichung 402367, Taiwan; cshy352@csh.org.tw; 2 Department of Family and Community Medicine, Chung Shan Medical University Hospital, Taichung 402367, Taiwan; 3 Department of Nutrition, Chung Shan Medical University, Taichung 402367, Taiwan; ccrazybon@gmail.com (P.-S.C.); sunny850209@gmail.com (C.-J.C.); yuyunlawliet@gmail.com (Y.-Y.H.); 4 Graduate Program in Nutrition, Chung Shan Medical University, Taichung 402367, Taiwan; 5 Department of Nutrition, Chung Shan Medical University Hospital, Taichung 402367, Taiwan

**Keywords:** β-carotene, osteoarthritis, obesity, metabolic syndrome, inflammation

## Abstract

This study was conducted to investigate the β-carotene status in osteoarthritis (OA) patients and examine its relationships with the risk of inflammation and metabolic syndrome. OA patients were stratified by obesity based on body fat percentage (obese OA, *n* = 44; non-obese OA, *n* = 56), and sixty-nine subjects without OA or obesity were assigned as a non-obese control group. β-carotene, metabolic parameters, and inflammation status were assessed. Obese OA patients exhibited a significantly higher rate of metabolic syndrome (*p* = 0.02), abdominal obesity (*p* < 0.01), and lower β-carotene status (*p* < 0.01) compared with non-obese OA and non-obese controls. After adjusting for potential confounders, β-carotene status (≥0.8 µM) was significantly inversely correlated with the risk of metabolic syndrome (odds ratio = 0.27, *p* < 0.01), abdominal obesity (odds ratio = 0.33, *p* < 0.01), high blood pressure (odds ratio = 0.35, *p* < 0.01), hyperglycemia (odds ratio = 0.45, *p* < 0.05), and inflammation (odds ratio = 0.30, *p* = 0.01). Additionally, subjects who had a high β-carotene status with a low proportion of metabolic syndrome when they had a low-grade inflammatory status (*p* < 0.01). Obese OA patients suffered from a higher prevalence of metabolic syndrome and lower β-carotene status compared to the non-obese controls. A better β-carotene status (≥0.8 µM) was inversely associated with the risk of metabolic syndrome and inflammation, so we suggest that β-carotene status could be a predictor of the risk of metabolic syndrome and inflammation in patients with and without OA.

## 1. Introduction

Osteoarthritis (OA) is a disease that may cause joint dysfunction and physical disability in individuals during aging [1]. Risk factors for OA include aging, gender, genetic variants, and obesity [2]. Among these risk factors, obesity is one of the acquired risk factors for OA that may be related to increased stress on the tibiofemoral cartilage [3]. Many studies have found that OA patients have a higher prevalence of obesity [4,5,6]. Obesity may induce metabolic disorders, such as hypertension, dyslipidemia, and hyperglycemia, and cause the burden of OA [7,8]. These metabolic disorders may result in an increased inflammatory status, and mediate the unfavorable progression of OA [9,10].

Recently, studies have indicated that nutrients with antioxidant capacity may have effects on the inflammatory response and metabolic syndrome [11,12]. β-carotene is a natural molecule and the most abundant provitamin A carotenoid in foods [13]. β-carotene is known to be highly absorbed and utilized in humans [13]. Studies have found that β-carotene acts as an antioxidant to protect against cardiovascular disease [14], type 2 diabetes, and obesity [15]. A recent systematic review and meta-analysis concluded that β-carotene status was inversely associated with metabolic syndrome [16]. Oxidative stress may be a result of the progression of the diseases; therefore, studies have found that OA patients suffer from a depletion of antioxidant nutrients, such as vitamin C and vitamin E [17,18]. However, there are few studies examining the level of β-carotene in OA patients. Thus, the purpose of this study was to investigate β-carotene status in OA patients and examine its relationships with the risk of inflammation and metabolic syndrome.

## 2. Materials and Methods

### 2.1. Subjects

One hundred OA patients were recruited for this study. OA was diagnosed by a standing anterior-to-posterior knee X-ray examination with associated exam results for a Kellgren and Lawrence grade greater than or equal to 2 [19]. We further stratified OA patients with obesity according to body fat percentage, which was measured by dual-energy X-ray absorptiometry (DXA). Body fat percentage ≥25% for male or ≥30% for female was defined as obesity [20]. Forty-four OA patients with obesity and fifty-six OA patients without obesity were assigned to as obese OA and non-obese OA groups, respectively. Additionally, sixty-nine subjects without OA or obesity were assigned to the non-obese control group. The exclusion criteria of the subjects were as follows: (1) patients with rheumatoid arthritis; (2) the use of glucosamine sulfate, a non-steroidal anti-inflammatory drug, or hyaluronic acid injection therapy in the past month; (3) the use of nutritional supplements; and (4) knee replacement surgery. This study was approved by the Institutional Review Board of Chung Shan Medical University Hospital, Taiwan (CSMUH No: CS2-17095). Each subject provided written informed consent to participate in the study.

### 2.2. Characteristics and Anthropometric Assessments

Data on the basic characteristics of the subjects, including age, gender, smoking cigarette, alcohol use, exercise, and medications, were collected by a questionnaire and medical records. Blood pressure was measured by a digital electronic sphygmomanometer (Hartmann Tensoval^®^ duo control, Heidenheim, Germany). Anthropometric assessments were as follows: height and body weight were measured by a height meter (Jeng-Jyi M-150 L, Changhua, Taiwan) and a weight scale (Tanita BC-545N, Akita, Japan) and were used to calculate the body mass index (BMI; kg/m^2^). Waist circumference was measured using a tape. Body fat percentage, visceral fat mass, whole and trunk muscle mass, whole skeletal muscle mass index, and appendicular skeletal muscle mass index were measured using a DXA machine (Hologic, ASY-05119, Marlborough, MA, USA).

### 2.3. Blood Collection and Biochemical Measurements

Vacutainer blood collection tubes with anticoagulant (K_2_-EDTA or sodium fluoride) or without anticoagulant were used to collect fasting venous blood samples. Plasma and serum samples were prepared for β-carotene and biochemical measurements, respectively, after centrifugation at 4 °C and 3000 rpm for 15 min. The level of fasting glucose and lipid profiles, including total cholesterol (TC), low-density lipoprotein cholesterol, high-density lipoprotein cholesterol (HDL-C), and triglycerides, were measured by an automated chemistry analyzer (Beckman Coulter, DXC 800, Brea, CA, USA), and the level of high-sensitivity C reactive protein (hs-CRP) was measured by an automatic clinical analyzer (Hitachi 7600-110, Tokyo, Japan).

### 2.4. β-Carotene Status Measurement

Level of β-carotene was measured by high-performance liquid chromatography (HPLC) with an ultraviolet detector [21] under yellow light to prevent photo-destruction. The standard of all-trans-β-carotene was obtained from Sigma-Aldrich (Merck, Darmstadt, Germany). The protein in plasma was precipitated by cold ethanol and cold hexane (containing 1 g/L of butylated hydroxytoluene) and was then centrifuged. The supernatant was filtered for HPLC analysis. The mobile phase was a mixture of methanol and ethanol. The analysis column was a Purospher^®^ STAR RP-18 (Merck, Darmstadt, Germany) and the wavelength was set at 450 nm. The flow rate was kept constant at 1.0 mL/min. The lower limit of detection was 0.05 µM. The mean analytical recovery of β-carotene was 102%. The mean intra- and inter-assay coefficients of variability for β-carotene were 3.9% and 4.6%, respectively.

### 2.5. Metabolic Syndrome and Inflammation Status

The diagnosis of metabolic syndrome was made according to the Health Promotion Administration, Ministry of Health and Welfare in Taiwan [22], and five components of metabolic syndrome were defined as follows: abdominal obesity was defined as waist ≥90 cm for male and ≥80 cm for female; high blood pressure was defined as systolic blood pressure (SBP) ≥ 130 mmHg and diastolic blood pressure (DBP) ≥ 85 mmHg (including use of antihypertensive drugs); hyperglycemia was defined as fasting glucose ≥ 5.55 mmol/L (including use of hypoglycemic agents); hypertriglyceridemia was defined as the level of triglycerides ≥ 1.70 mmol/L (including use of hypolipidemic agents); low HDL-C was defined as HDL-C ≤ 1.04 mmol/L in males and ≤1.30 mmol/L in females. Subjects with metabolic syndrome greater than or equal to three components were diagnosed with metabolic syndrome. Regarding the inflammation status, a level of hs-CRP ≥ 3.0 mg/L was defined as high inflammation, and hs-CRP ≥ 1.0 mg/L was defined as low-grade inflammation status according to previous studies [23,24].

### 2.6. Statistical Analyses

We used SigmaPlot software (version 12.0, Systat, San Jose, CA, USA) to conduct all statistical tests in the present study. Continuous variables are shown as the mean ± standard deviation (median), while categorical variables are shown as percentages. We used the Shapiro–Wilk test to examine the normality of the distribution of the data. The differences among the three groups in characteristics and β-carotene status were examined using one-way ANOVA or Kruskal–Wallis test; post hoc tests were further used to compare differences among the groups. The differences between the two groups (stratified by disease groups) in β-carotene status were examined using an independent t-test or Mann–Whitney rank sum test. The differences in categorical variables were examined by using a Chi-square test or Fisher’s exact test. Spearman’s rank order correlation coefficient was used to examine the correlations between β-carotene status and metabolic syndrome and its components and inflammatory status. Multiple logistic regression analyses were used to examine the correlations between β-carotene status and the risk of metabolic syndrome and its components and inflammation after adjusting for potential confounders. Statistical significance was indicated when *p* values were < 0.05.

## 3. Results

### 3.1. Characteristics of the Subjects

The characteristics of the subjects in the present study are shown in Table 1. Obese OA subjects had significantly higher ages (*p* < 0.01), BMIs (*p* < 0.01), visceral fat mass (*p* < 0.01), prevalence of metabolic syndrome (*p* = 0.02), abdominal obesity (*p* < 0.01), and lower trunk muscle mass (*p* = 0.02) than non-obese OA and non-obese controls and were disproportionally female (*p* < 0.01). Significantly higher values for waist (*p* < 0.01) and systolic blood pressure (*p* < 0.05), as well as lower whole body muscle mass (*p* = 0.02), were also found in obese OA than those in the non-obese controls group. In addition, a slightly higher level of hs-CRP was found in obese OA subjects than in the other groups (*p* = 0.09).

### 3.2. β-Carotene Status

**Figure 1:**
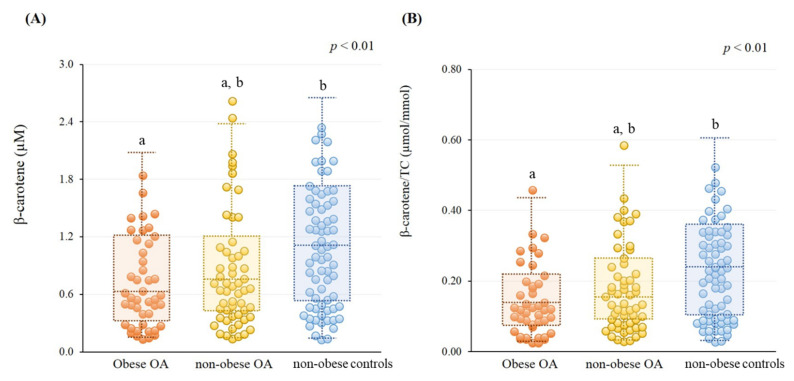
β-carotene status in subjects. (**A**) Level of β-carotene status (obese OA, 0.70 ± 0.46 µM; non-obese OA, 0.85 ± 0.62 µM; non-obese controls, 1.05 ± 0.61 µM). (**B**) Level of β-carotene/TC (obese OA, 0.14 ± 0.10 µmol/mmol; non-obese OA, 0.17 ± 0.12 µmol/mmol; non-obese controls, 0.22 ± 0.13 µmol/mmol). ^a,b^ The significant difference was examined by post hoc test, *p* < 0.05. OA, osteoarthritis; TC, total cholesterol.

Figure 1 shows the β-carotene status in subjects. Overall, OA patients had a significantly lower β-carotene status than the non-obese controls group (OA vs. non-obese controls, β-carotene, 0.78 ± 0.56 µM vs. 1.05 ± 0.61 µM, *p* < 0.01; β-carotene/TC, 0.16 ± 0.11 µmol/mmol vs. 0.22 ± 0.13 µmol/mmol, *p* < 0.01). Obese OA subjects exhibited a significantly lower level of β-carotene (Figure 1A, obese OA vs. non-obese controls, 0.70 ± 0.46 µM vs. 1.05 ± 0.61 µM, *p* < 0.01) and β-carotene/TC (Figure 1B, obese OA vs. non-obese controls, 0.14 ± 0.10 µmol/mmol vs. 0.22 ± 0.13 µmol/mmol, *p* < 0.01) than the non-obese controls group. However, there was no significant difference in β-carotene status between the non-obese OA and non-obese control groups, as well as obese OA and non-obese OA. There was a higher rate of β-carotene deficiency (β-carotene < 0.3 μM) in obese OA than other groups (obese OA, 27.3%, non-obese OA, 16.1%, non-obese controls, 7.2%, *p* = 0.02). In addition, we examined the difference of the same age and gender among the three groups. The results showed that obese OA patients who were elderly (age ≥ 65 years, obese OA vs. non-obese controls, β-carotene, 0.72 ± 0.48 µM vs. 1.28 ± 0.57 µM, *p* < 0.01; β-carotene/TC, 0.14 ± 0.10 µmol/mmol vs. 0.26 ± 0.11 µmol/mmol, *p* < 0.01) or female (obese OA vs. non-obese controls, β-carotene, 0.73 ± 0.46 µM vs. 1.23 ± 0.60 µM, *p* < 0.01; β-carotene/TC, 0.15 ± 0.10 µmol/mmol vs. 0.24 ± 0.11 µmol/mmol, *p* < 0.01) exhibited a significantly lower β-carotene status than those in the non-obese controls.

### 3.3. Correlations between β-Carotene Status and Metabolic Syndrome and Inflammation

Correlations between β-carotene status and the proportion of participants with metabolic syndrome and its components and inflammation are shown in Table 2. β-carotene level and β-carotene/TC were significantly inversely associated with the proportion of those with metabolic syndrome (*p* < 0.05), abdominal obesity (*p* < 0.05), high blood pressure (*p* < 0.05), and inflammation (*p* < 0.05) in OA patients, as well as in all subjects (*p* < 0.05). In the non-obese controls group, there was an inverse association between β-carotene status and the proportion of those with metabolic syndrome (*p* < 0.05) and abdominal obesity (*p* < 0.05).

**Figure 2:**
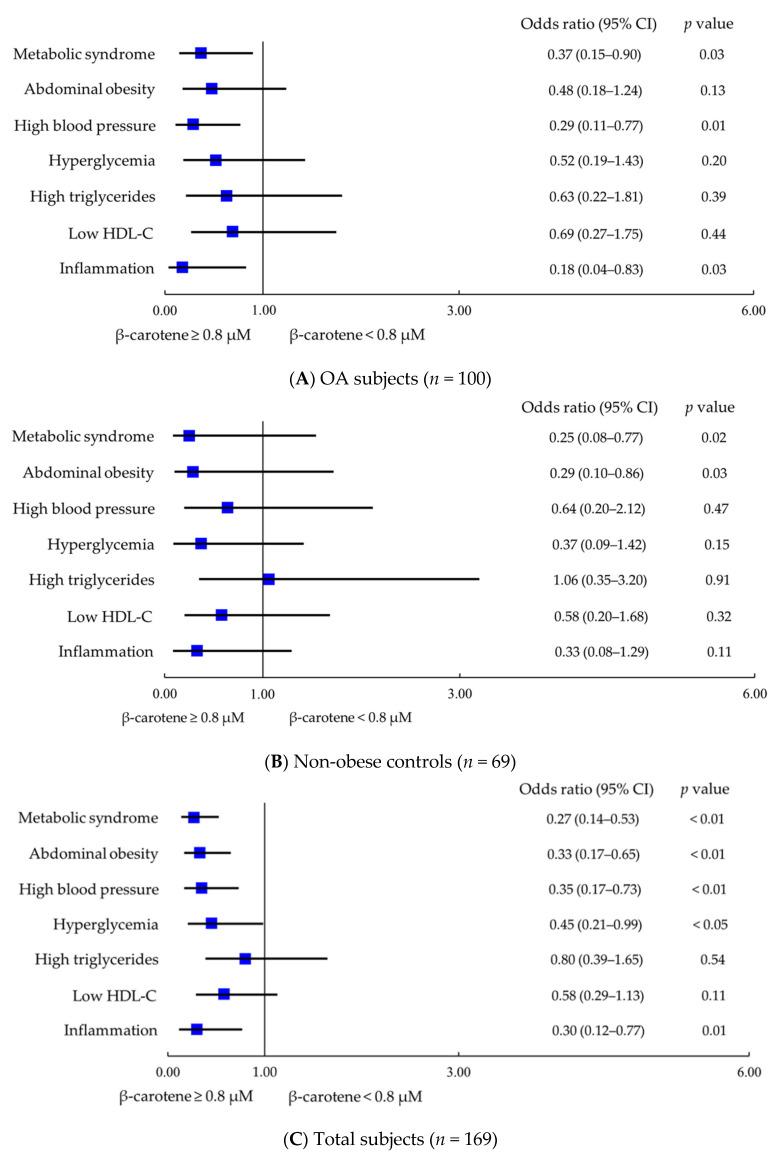
β-carotene status and the risk of metabolic syndrome and inflammation. (**A**) Correlations between β-carotene status and the risk of metabolic syndrome and inflammation in OA subjects after adjusting for age, gender, and obesity. (**B**) Correlations between β-carotene status and the risk of metabolic syndrome and inflammation in non-obese controls after adjusting for age and gender. (**C**) Correlations between β-carotene status and the risk of metabolic syndrome and inflammation in all subjects after adjusting for age, gender, obesity and disease. CI, confidence interval; HDL-C, high-density lipoprotein cholesterol; OA, osteoarthritis.

Furthermore, we calculated the odds ratios for metabolic syndrome and its components, as well as inflammation according to the level of β-carotene in OA patients (Figure 2A) non-obese OA (Figure 2B), and all subjects (Figure 2C). After adjusting for age and gender and obesity, OA subjects with a higher level of β-carotene (≥0.8 µM) had a significantly inverse association with the risk of metabolic syndrome (odds ratios: 0.37, *p* = 0.03), high blood pressure (odds ratios: 0.29, *p* = 0.01), and inflammation (odds ratios: 0.18, *p* = 0.03). In non-obese control group, subjects who had a higher level of β-carotene (≥0.8 µM) had a significantly inverse association with the risks of metabolic syndrome (odds ratios: 0.25, *p* = 0.02) and abdominal obesity (odds ratios: 0.29, *p* = 0.03) after adjusting for age and gender. In all subjects, after adjusting for age and gender, obesity and disease, subjects with a higher level of β-carotene (≥0.8 µM) not only had a significantly inverse association with the risk of metabolic syndrome (odds ratios: 0.27, *p* < 0.01), high blood pressure (odds ratios: 0.35, *p* < 0.01) and inflammation (odds ratios: 0.30, *p* = 0.01), but also had an inversely association with the risk of abdominal obesity (odds ratios: 0.33, *p* < 0.01) and hyperglycemia (odds ratios: 0.45, *p* < 0.05).

### 3.4. Effect of β-Carotene and Inflammatory Status on Metabolic Syndrome

**Figure 3:**
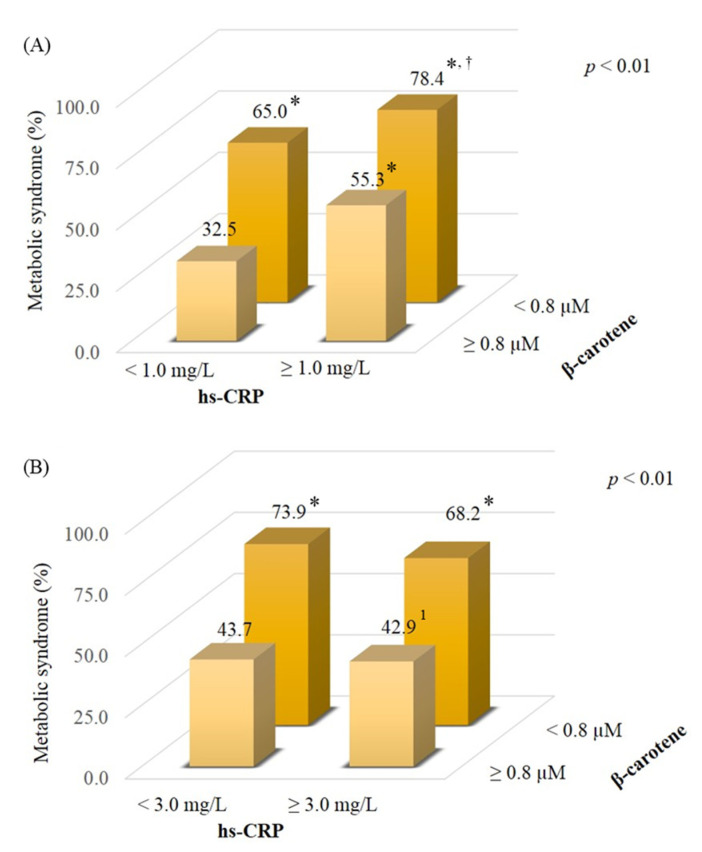
Effects of β-carotene and inflammation status on metabolic syndrome. (**A**) The proportions of those with metabolic syndrome according to the levels of β-carotene and a low-grade inflammatory status (hs-CRP ≥ 1.0 mg/L). (**B**) The proportions of those with metabolic syndrome according to the levels of β-carotene and a high inflammatory status (hs-CRP ≥ 3.0 mg/L). ^1^ Value compared with other groups (*p* > 0.05). * Value compared with the level of β-carotene ≥ 0.8 µM with hs-CRP < 1.0 or 3.0 mg/L, *p* < 0.05. ^†^ Value was compared with the level of β-carotene ≥ 0.8 µM with hs-CRP ≥ 1.0 mg/L, *p* < 0.05. hs-CRP, high-sensitivity C reactive protein.

Figure 3 shows the proportion of subjects with metabolic syndrome stratified by levels of β-carotene and hs-CRP. Subjects who had a lower β-carotene status (<0.8 µM) had a high rate of metabolic syndrome compared to those who had a better β-carotene status (≥0.8 µM) regardless of inflammation status (Figure 3A, 78.4% vs. 32.5%, *p* < 0.01; 65.0% vs. 32.5%, *p* < 0.01). In addition, the proportion of participants with metabolic syndrome was lower in subjects who had a better β-carotene status with a low-grade inflammatory status (hs-CRP ≥ 1.0 mg/L) than those with a lower β-carotene status (55.3% vs. 78.4%, *p* = 0.02). Similar data were also shown to have a high inflammatory status (Figure 3B, hs-CRP ≥ 3.0 mg/L). A high rate of metabolic syndrome was found in the subjects who had a lower β-carotene status compared to those with a better β-carotene status (Figure 3B, 73.9%, vs. 43.7%, *p* < 0.01; 68.2% vs. 43.7%, *p* < 0.05).

## 4. Discussion

This study is the first to explore β-carotene status in OA patients. β-carotene has been demonstrated to prevent oxidative stress and mediate inflammation in metabolically dysfunctional adipocytes [25,26]. Comprehensive data from carotenoid intake and blood or tissue concentration studies indicated that the normal ranges for plasma β-carotene are 0.3–0.9 µM [27]. In our study, the level of β-carotene in OA or non-obese control subjects seemed to be within the normal reference range, but we noted that patients with OA and obese OA subjects had a significantly lower β-carotene status than the non-obese control group (Figure 1), and the obese OA group had a higher rate of β-carotene deficiency (β-carotene < 0.3 μM) than other groups. In the present study, we found that our OA patients may suffer from a lower β-carotene status due to the disease and metabolic disorders. As we found that β-carotene status was significantly inversely correlated with metabolic syndrome and inflammation status (Table 2), we tried to characterize a better β-carotene status to influence the risk of metabolic syndrome and inflammation. We found that a better β-carotene status was ≥ 0.8 μM. This level of β-carotene was based on the median level of all subjects. As OA patients with a better β-carotene status (≥0.8 μM) exhibited a significantly inverse association with the risk of metabolic syndrome and inflammation, the same trend was also found in all subjects (Figure 2). A cross-sectional investigation in a community-based study in Japan found that there was no association between β-carotene status and OA, but the risk for OA was related to a low β- and γ-tocopherols status [28]. In the study [28], we noted that non-OA participants, who were rural Japanese inhabitants, who smoked and drank a significant amount may affect β-carotene status, resulting in no significant difference between the OA and non-OA groups. In our study, there was no significant difference in smoking and drinking habits among the groups, but obese OA subjects in the present study seem to suffer from a high prevalence of metabolic syndrome (Table 1), which may lead to β-carotene depletion. Since metabolic syndrome is related to inflammatory status [9], we further explored β-carotene status in subjects suffering from both metabolic syndrome and inflammation status. We found a high rate of metabolic syndrome in subjects who had a lower β-carotene status (<0.8 μM), especially those who suffered from a low-grade inflammatory status (hs-CRP ≥ 1.0 mg/L); on the contrary, the proportion of those with metabolic syndrome was significantly lower for subjects who had a better β-carotene status (≥0.8 μM) (Figure 3). Therefore, considering metabolic status, intervention with carotenoid-rich foods (a variety of fruits and vegetables) could be advised in OA to improve their β-carotene status, particularly in obese OA, which may benefit the prevention of metabolic syndrome and inflammation.

Obesity may have dual effects on OA progression and is a component of metabolic syndrome, especially central obesity [29,30]. Data from the Third National Health and Nutrition Examination Survey (NHANES III) in the U.S.A. found that metabolic syndrome had a prevalence of 59% in the OA population and 23% of the population without OA [31]. Among the five components of metabolic syndrome, 75% and 63% of the OA population suffered from high blood pressure and abdominal obesity, respectively, and these proportions were higher than in those without OA [31]. In the present study, we used a DXA machine to measure total body fat to stratify OA patients with obesity. A total of 86.4% of obese OA belonged to abdominal obesity, which was significantly higher than that of the other groups (Table 1). It is necessary to pay attention to reducing the risk of abdominal obesity because it implies visceral fat accumulation, which can lead to the development of type 2 diabetes and cardiovascular diseases [32,33]. In this study, we noted that β-carotene status was significantly inversely correlated with the proportion of those with abdominal obesity in OA patients (Table 2). Although there was no significantly negative association with the risk of abdominal obesity in OA patients with a better β-carotene status (≥0.8 μM) (Figure 2A), we found that a better β-carotene status was significantly inversely associated with the risk of abdominal obesity in all subjects after adjusting for potential confounders (Figure 2C). Recently, a systematic review and meta-analysis report showed a low carotenoids status, such as β-carotene, was a risk factor for overweight and obesity in a healthy population [34]. Similar findings from the NHANES III in the USA indicated low micronutrient levels (including β-carotene) among the individuals who were obese [35]. Two studies from a healthy Japanese population found that obesity was associated with a decreased level of β-carotene [36,37], especially in abdominal obesity [37]. Most of the studies investigated the correlation of obesity or overweight according to the value of BMI [34,35,36,37]. In this study, we defined obesity according to the total body fat percentage measured by DXA. Using the body fat percentage determine obesity was more accurate than using BMI. In the results of Table 2, we found a significant inverse association between β-carotene status and abdominal obesity and total body fat obesity in all subjects. Thus, we support that β-carotene status was associated with obesity, particularly abdominal obesity. It has been indicated that β-carotene has antiobesity effects [38] because it is a precursor to apocarotenoids and regulates adipocyte physiology through suppression of peroxisome-proliferator-activated receptor (PPAR)-γ, reducing body adiposity [39,40,41,42]. Based on the results, we suggest that β-carotene status could be a predictor of the risk of obesity in OA.

In addition to abdominal obesity, we also noted that more than 80% of obese OA patients suffered from high blood pressure and hyperglycemia, although the proportions were not significantly different between the other two groups (Table 1). In the results of the present study, we found that the β-carotene level was significantly inversely correlated with high blood pressure and hyperglycemia (Table 2). Subjects with a better β-carotene status may have an inverse association with the risk of high blood pressure and hyperglycemia (Figure 2). Data from the National Health and Nutrition Examination Survey 2007–2014 found that dietary β-carotene intake was inversely associated with hypertension in US adults [43]. β-carotene has also been demonstrated to play a preventive role in type 2 diabetes [15,44]. β-carotene modulates blood pressure and the metabolism of blood glucose due to its antioxidant capacity. β-carotene may alleviate endothelial cell dysfunction due to oxidative damage [44,45,46] and regulate the functions of pancreatic β-cells against insulin resistance [47,48]. In fact, OA is not only an oxidative stress-related disease but is also associated with metabolic status [49]. OA combined with obesity or metabolic syndrome may worsen the progression of the disease. Based on the results of this study, we found that β-carotene was inversely associated with the risk of metabolic disorders; thus, OA patients should monitor their β-carotene status, particularly those with metabolic syndrome.

This study is the first clinical study to investigate the β-carotene status in patients with OA and its correlations with metabolic syndrome, its components, and inflammation. However, we cannot clarify the cause and effect between β-carotene and inflammation or metabolic abnormalities through this cross-sectional study. Additionally, we defined obesity by measuring body fat in the present study. The BMI cutoff point is commonly used to define obesity due to its high specificity; however, BMI is less accurate in identifying adiposity [20,50]. Since excessive body fat is associated with metabolic disorders, it should be considered as a measure of obesity in patient care settings. Future research should explore the effect of β-carotene supplementation on the anti-inflammatory and metabolic status of obese OA patients.

## 5. Conclusions

Obese OA patients suffer from a higher prevalence of metabolic syndrome and a lower β-carotene status compared to non-obese controls. Better β-carotene status (≥0.8 µM) was significantly inversely correlated with the risk of metabolic syndrome and inflammation; therefore, we suggest that β-carotene status could be a predictor of the risk of metabolic syndrome and inflammation in patients with and without OA. 

## Figures and Tables

**Table 1:** Characteristics of subjects.

Characteristics	Obese OA ^2^ (*n* = 44)	Non-Obese OA (*n* = 56)	Non-Obese Controls (*n* = 69)	*p* Value
Age (years)	72.0 ± 8.7 (73.0) ^1,a^	67.5 ± 9.5 (68.0) ^b^	60.4 ± 9.6 (60.0) ^c^	<0.01
Female (*n*, %)	38 (86.4%) ^a^	35 (62.5%) ^b^	33 (47.8%) ^b^	<0.01
BMI (kg/m^2^)	26.6 ± 3.3 (26.9) ^a^	24.3 ± 3.8 (24.0) ^b^	23.8 ± 4.2 (23.7) ^b^	<0.01
Waist (cm)	90.2 ± 7.9 (90.9) ^a^	86.1 ± 11.2 (86.2) ^a,b^	83.7 ± 11.7 (82.8) ^b^	<0.01
Visceral fat mass (g)	607.8 ± 159.2 (621.0) ^a^	414.9 ± 192.8 (412.5) ^b^	394.4 ± 185.6 (380.5) ^b^	<0.01
Whole body muscle mass (kg)	39.4 ± 6.4 (38.7) ^a^	43.8 ± 10.1 (42.2) ^a,b^	45.3 ± 11.6 (44.3) ^b^	0.02
Trunk muscle mass (kg)	20.1 ± 3.6 (19.4) ^a^	22.4 ± 5.0 (22.1) ^b^	22.7 ± 5.6 (21.9) ^b^	0.02
WSMI (kg/m^2^)	16.5 ± 2.2 (16.4)	17.3 ± 2.9 (17.3)	17.4 ± 3.1 (17.3)	0.30
ASMI (kg/m^2^)	6.8 ± 1.0 (6.9)	7.2 ± 1.4 (7.1)	7.4 ± 1.6 (7.4)	0.11
SBP (mmHg)	139.3 ± 19.5 (137.0) ^a^	133.6 ± 19.2 (133.0) ^a,b^	130.6 ± 16.1 (129.0) ^b^	<0.05
DBP (mmHg)	80.3 ± 12.5 (78.5)	80.8 ± 10.2 (80.0)	82.9 ± 13.0 (84.0)	0.46
Fasting glucose (mmol/L)	6.5 ± 1.2 (6.1)	6.4 ± 1.2 (6.2)	6.2 ± 1.3 (5.8)	0.33
Triglycerides (mmol/L)	1.4 ± 0.7 (1.2)	1.2 ± 0.7 (1.0)	1.5 ± 0.8 (1.3)	0.11
HDL-C (mmol/L)	1.4 ± 0.3 (1.3)	1.4 ± 0.4 (1.3)	1.3 ± 0.4 (1.2)	0.43
LDL-C (mmol/L)	3.2 ± 1.0 (3.0)	2.9 ± 0.7 (2.9)	3.0 ± 0.7 (2.9)	0.28
Total cholesterol (mmol/L)	5.2 ± 1.1 (4.9)	4.9 ± 0.8 (5.0)	5.0 ± 0.9 (4.9)	0.71
Hs-CRP (mg/L)	1.8 ± 1.7 (1.4)	3.0 ± 9.5 (1.1)	1.8 ± 3.5 (0.9)	0.09
Metabolic syndrome (%) ^3^	34 (77.3%) ^a^	29 (51.8%) ^b^	37 (53.6%) ^b^	0.02
Abdominal obesity (%) ^4^	38 (86.4%) ^a^	34 (60.7%) ^b^	32 (46.4%) ^b^	<0.01
High blood pressure (%) ^5^	37 (84.1%)	39 (69.6%)	49 (71.0%)	0.20
Hyperglycemia (%) ^6^	38 (86.4%)	42 (75.0%)	54 (78.3%)	0.37
High triglycerides (%) ^7^	10 (22.7%)	12 (21.4%)	22 (31.9%)	0.35
Low HDL-C (%) ^8^	20 (45.5%)	15 (26.8%)	26 (37.7%)	0.15
Inflammation (%) ^9^	6 (13.6%)	11 (19.6%)	12 (17.4%)	0.73
Lifestyle				
Smokes cigarettes				0.17
Current	0 (0.0%)	1 (1.8%)	3 (4.3%)	
Ever	5 (11.4%)	7 (12.5%)	16 (23.2%)	
Never	39 (88.6%)	48 (85.7%)	50 (72.5%)	
Alcohol use				0.09
Current	0 (0.0%)	5 (8.9%)	7 (10.1%)	
Ever	1 (2.3%)	3 (5.4%)	7 (10.1%)	
Never	43 (97.7%)	48 (85.7%)	55 (79.7%)	
Exercise	22 (50.0%)	30 (53.6%)	39 (56.5%)	0.79

^1^ means ± SD (medians). ^2^ Obese: a body fat percentage ≥25% in males and ≥30% in females. ^3^ Subjects who had more than three components (high blood pressure, abdominal obesity, low HDL-C, high triglycerides, and hyperglycemia) were defined as having metabolic syndrome. ^4^ Abdominal obesity is defined as waist ≥ 90 cm for males and ≥80 cm for females. ^5^ High blood pressure is defined as SBP ≥ 130 mmHg and DBP ≥ 85 mmHg (including use of antihypertensive drugs). ^6^ Hyperglycemia is defined as the level of fasting glucose ≥ 5.55 mmol/L (including use of hypoglycemic agents). ^7^ High triglycerides is defined as the level of triglycerides ≥ 1.70 mmol/L (including use of hypolipidemic agents). ^8^ Low HDL-C is defined as the level of HDL-C ≤ 1.04 mmol/L for males and ≤ 1.30 mmol/L for females. ^9^ Inflammation is defined as the level of Hs-CRP ≥ 3.0 mg/L. ^a–c^ The significant difference was examined by post hoc test, *p* < 0.05. ASMI, appendicular skeletal muscle mass index; BMI, body mass index; DBP, diastolic blood pressure; HDL-C, high-density lipoprotein cholesterol; Hs-CRP, high-sensitivity C reactive protein; LDL-C, low-density lipoprotein cholesterol; OA, osteoarthritis; SBP, systolic blood pressure; WSMI, whole skeletal muscle mass index.

**Table 2:** Correlations between β-carotene status and metabolic syndrome and its components and inflammation.

Parameters	β-Carotene (µM)	β-Carotene/TC (µmol/mmol)
	*r* ^1^ (*p* value)	
OA subjects (*n* = 100)		
Metabolic syndrome (%)	−0.21 (0.03)	−0.21 (0.03)
Abdominal obesity (%)	−0.24 (0.02)	−0.22 (0.03)
High blood pressure (%)	−0.24 (0.02)	−0.28 (<0.01)
Hyperglycemia (%)	−0.16 (0.12)	−0.12 (0.23)
High triglycerides (%)	−0.07 (0.50)	−0.13 (0.20)
Low HDL-C (%)	−0.09 (0.40)	−0.04 (0.70)
Inflammation (%)	−0.23 (0.02)	−0.18 (0.07)
Total body fat obesity (%)	−0.10 (0.31)	−0.14 (0.18)
Non-obese controls (*n* = 69)		
Metabolic syndrome (%)	−0.35 (<0.01)	−0.36 (<0.01)
Abdominal obesity (%)	−0.22 (0.07)	−0.27 (0.03)
High blood pressure (%)	−0.12 (0.32)	−0.11 (0.36)
Hyperglycemia (%)	−0.14 (0.25)	−0.10 (0.41)
High triglycerides (%)	−0.08 (0.53)	−0.16 (0.20)
Low HDL-C (%)	−0.08 (0.52)	−0.04 (0.75)
Inflammation (%)	−0.22 (0.08)	−0.22 (0.07)
Total body fat obesity (%) ^2^	No analysis ^2^	No analysis ^2^
Total subjects (*n* = 169)		
Metabolic syndrome (%)	−0.30 (<0.01)	−0.29 (<0.01)
Abdominal obesity (%)	−0.29 (<0.01)	−0.29 (<0.01)
High blood pressure (%)	−0.20 (<0.01)	−0.21 (<0.01)
Hyperglycemia (%)	−0.16 (0.04)	−0.12 (0.11)
High triglycerides (%)	−0.05 (0.52)	−0.10 (0.20)
Low HDL-C (%)	−0.09 (0.24)	−0.04 (0.58)
Inflammation (%)	−0.22 (<0.01)	−0.20 (0.01)
Total body fat obesity (%)	−0.18 (0.02)	−0.20 (<0.01)

^1^ Correlation coefficient. ^2^ Subjects in the non-obese controls without total body fat obesity. HDL-C, high-density lipoprotein cholesterol; OA, osteoarthritis; TC, total cholesterol.

## Data Availability

The data presented in this study are available on request from the corresponding author.
